# Community Perceptions of Accessing Surgical and Anesthetic Care in Rural Guatemala: A Cross-Sectional Survey

**DOI:** 10.7759/cureus.85783

**Published:** 2025-06-11

**Authors:** Jakob E Gamboa, Antonio G Bolaños, Colby G Simmons

**Affiliations:** 1 Anesthesiology, University of Colorado School of Medicine, Aurora, USA; 2 Community Health, Fundación para la Salud Integral de los Guatemaltecos, Quetzaltenango, GTM

**Keywords:** access to care, anesthesia, community health, global health, low- and middle-income country

## Abstract

Access to essential surgical and anesthesia care is limited in rural areas in low- and middle-income countries. Knowledge of the perceptions of access and utilization of surgical care among rural populations in Guatemala is lacking. This cross-sectional study examines self-reported trends and barriers to accessing surgical and anesthesia care in rural Southwest Guatemala. Semi-structured interviews were conducted with individuals who presented to the Trifinio Center for Human Development (TCHD) in Southwestern Guatemala and consented to participate. Information regarding household experience, community trends, and perceptions of access was recorded and analyzed for themes. Individuals from 50 different households were interviewed. There were 29 (58%) respondents who reported a prior surgical history in the household. The most commonly perceived procedures in this region were Cesarean section and appendectomy, reported by 35 (70%) and 38 (56%) households, respectively. Forty (80%) households described an overall preference within their communities for birth in a hospital setting, as well as concerns about increasing rates of Cesarean sections. The median distance, in time traveled, required to travel to the nearest surgical hospital was 60 minutes (interquartile range 11.25 minutes), with a maximum time of five hours (n=1). The nearest urban centers for specialized surgical care reported were Quetzaltenango in 47 (94%) respondents (approximately two hours travel time) and Guatemala City in 14 (28%) respondents (approximately five hours travel time). Among respondents, 46 (92%) report inadequate access to surgical and anesthesia care in their communities. The primary perceived barriers to care were financial costs in 35 (70%) households, with reported out-of-pocket costs ranging from US$384.60 to US$1282.05, followed by geographic distance in 22 (44%) households and lack of quality of care in 12 (24%) households. In this study, we highlight a significant demand for essential surgeries in rural areas and identify perceived barriers to surgical and anesthesia care in Southwestern Guatemala. Future efforts should integrate community perspectives to ensure patient-centered approaches to improve access to essential care in marginalized populations.

## Introduction

The provision of surgery and anesthesia is essential to reducing the burden of surgical diseases and avoid preventable mortality and morbidity. Access to essential surgical, obstetric, and anesthesia care is limited in many low- and middle-income countries (LMICs) and disproportionately affects marginalized populations living in rural areas [1]. The barriers to care are multifactorial and are often related to problems with accessibility, availability, affordability, and acceptability [2].

Strategies to identify high-risk areas and gaps in care can help direct public health initiatives. Population-level data, such as metrics provided by the Organisation for Economic Co-Operation and Development (OECD), or surveys, such as the World Federation of Societies of Anesthesia (WFSA) Global Workforce Survey, provide valuable insight into practitioner density and specialization in a certain region [3]. Yet, although population-level data and health systems metrics provide valuable insight into regional care capabilities, hospital and workforce capacity data may only offer a partial view of a patient’s ability to receive care. Individual, socioeconomic, geographic, cultural, and other determinants may play a significant role in a person’s perceived access to care [4]. 

In Guatemala, workforce and resource constraints, lack of geographic access, as well as language barriers with indigenous communities, present significant challenges to providing health care to the large populations living in rural communities [5]. Of the 36 hospitals nationwide that provide surgical services, most of these facilities are located in large urban cities [6]. The perspectives of access to surgical services for communities in rural Southwest Guatemala are currently unknown. Therefore, the purpose of this study is to describe the perceived utilization and access to surgical, obstetric, and anesthesia care among these high-risk community members to inform public health efforts and reduce barriers to essential care.

## Materials and methods

We performed a cross-sectional study of community perceptions of access to surgical and anesthesia services in rural Southwest Guatemala. This article adheres to the applicable Enhancing the QUAlity and Transparency Of health Research (EQUATOR) Network guidelines for Strengthening the Reporting of Observational Studies in Epidemiology (STROBE) [7]. 

Study setting

**Figure 1:**
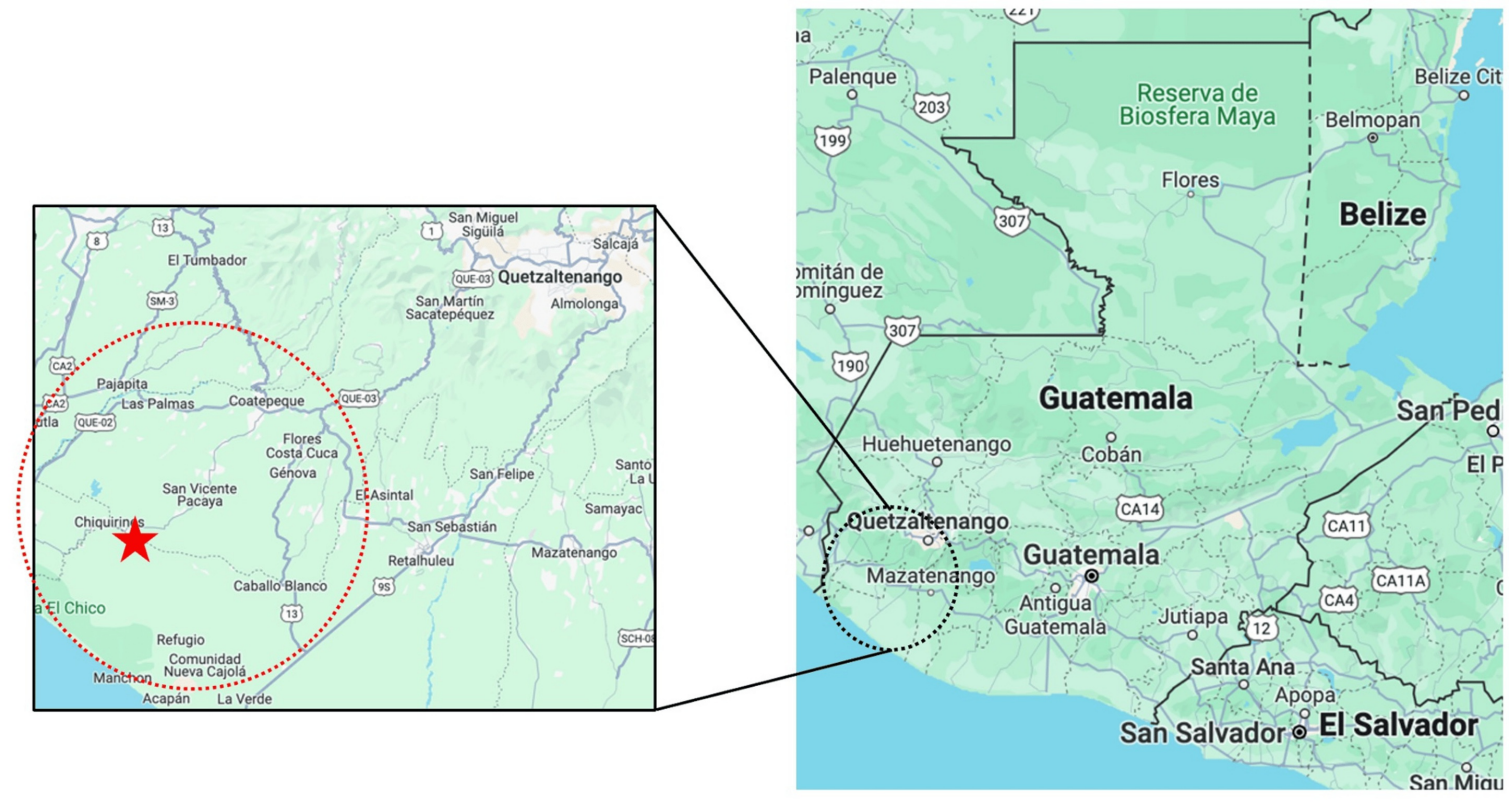
Original figure of a map of the Southwestern Trifinio region, Guatemala. TCHD identified with star. *Source*: Google Maps, retrieved 7/1/2024.

This study was performed at the Trifinio Center for Human Development (TCHD), which is located in the Southwest coastal lowlands of Guatemala, bordering the departments of San Marcos, Quetzaltenango, and Retalhuleu (Figure 1). This region is home to more than 30,000 individuals from 20 rural communities, with among the highest rates of poverty and poorest health outcomes in the country [8]. The TCHD is a collaborative health center that was developed to address the primary care, obstetric, and community health needs of these high-risk populations [9]. 

Study participants

In February 2023, an availability sample of individuals who presented to the TCHD was recruited to participate in the study. Patients presenting to the clinic were informed of the study by TCHD leadership. Individuals over the age of 18 who expressed interest and consented to participate were included in the study. Only individuals who refused to participate were excluded. 

Data collection

Eligible participants were interviewed by a language-certified research team member in Spanish, the preferred language of the region. Participants were informed that responses would remain confidential, would not affect the care received at the clinic, and that they could refuse to respond. Semi-structured interviews were performed through asking standardized questions regarding a history of surgery, community trends, distance to surgical centers, community childbirth preferences, and perceptions of barriers and access to surgery and anesthesia care, and then allowing participants to elaborate or discuss any other topics of interest or concern (Appendix 1). Among households who reported a history of surgery, additional questions about procedure type, location, and anesthesia method were requested, if they were willing to share. The gender of the primary respondents was recorded; no other identifiable data was collected. Only one individual per household was interviewed, and all responses were written by the research team. 

Data analysis

Interview responses were analyzed for themes and presented as frequencies and percentages of respondents. Descriptive data of the travel time to the nearest hospitals are presented as medians and interquartile ranges (IQR) due to the presence of outlier data. A formal sample size calculation was not performed. The sample size was based on the number of eligible households available during the designated study period, due to the availability of the research team to conduct interviews. This pragmatic approach was taken to maximize recruitment within the constraints of time and personnel, while reaching saturation of qualitative data and addressing study objectives.

Ethical considerations

This study received institutional review board exemption from the Colorado Multiple Institutional Review Board (#23-0129) and was approved and overseen by the local TCHD leadership. 

## Results

A total of 50 individuals from separate households from various communities were interviewed, of which 30 (60%) were female. A history of anesthesia care among household members was reported in 29 (58%) respondents, with Cesarean section (C-section) being the most common surgical procedure and reported by 17 (34%) households (Table 1). Neuraxial anesthesia was reportedly performed for 12 (70%) of the Cesarean sections.

**Table 1:** Interview questions and responses among participating households (total number = 50) N = number of households, C-section = Cesarean section, IQR = interquartile range.

Interview question	Response	N (%)
Has anyone in household received anesthesia?	Yes	29 (58%)
	No	21 (42%)
If willing to share, what surgery was performed?	Cesarean section	17/29 (59%)
	Appendectomy	5/29 (17%)
	Herniorrhaphy	3/29 (10%)
	Other	4/29 (14%)
Where was surgery performed?	Coatepeque	25/29 (86%)
	Quetzaltenango	2/29 (7%)
	Guatemala City	1/29 (3%)
	Other	1/29 (3%)
Method of anesthesia for household C-section?	Neuraxial	12/17 (71%)
	General anesthesia	4/17 (24%)
	Unsure	1/17 (6%)
What is the most common type of surgery in your community?	Cesarean section	35 (70%)
	Appendectomy	28 (56%)
	Cholecystectomy	12 (24%)
	Herniorrhaphy	11 (22%)
	Trauma/accident	4 (8%)
	Unknown	3 (6%)
	Other	6 (12%)
What is the closest surgical hospital?	Coatepeque	48 (96%)
	Unsure	2 (4%)
Travel time to closest surgical center (median)?	60 minutes (IQR 11.25)	-
Where would you go for emergency surgery?	Coatepeque	50 (100%)
Where would you go for specialized surgery?	Quetzaltenango	47 (94%)
	Guatemala City	14 (28%)
	Coatepeque (private hospital)	1 (2%)
Where do most women in your community deliver?	Hospital	40 (80%)
	Home (midwife-assisted)	5 (10%)
	Mixed	5 (10%)
How common is Cesarean section among all deliveries?	Common	44 (88%)
	Not common	4 (8%)
	Unsure	2 (4%)
Is there adequate access to anesthesia and surgery?	Yes	4 (8%)
	No	46 (92%)
What are the primary barriers to accessing care? (describe all that apply)	Cost	35 (70%)
	Distance/transport	22 (44%)
	Quality of care	12 (24%)
	Workforce/staffing	2 (4%)
	Delays in care	2 (4%)
	Fear	1 (2%)
	Missing/unknown	8 (16%)

**Figure 2:**
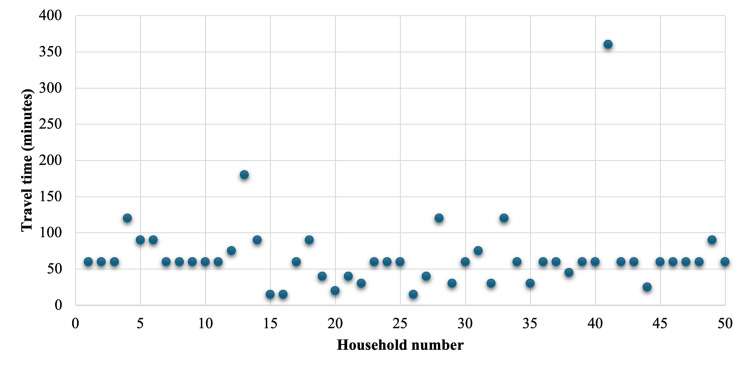
Estimated travel time to the nearest surgical center per household

The median distance, in time traveled, to the nearest surgical center was 60 minutes (IQR 11.25), with a maximum reported travel time of six hours (Figure 2). The nearest surgical hospital for most respondents was in the town of Coatepeque, Guatemala. The nearest reported urban centers for specialized surgical care were Quetzaltenango (approximately two-and-a-half to three hours from TCHD) and Guatemala City (approximately five to seven hours from TCHD). 

The most commonly perceived surgeries performed in their respective communities were C-section in 35 (70%) respondents, appendectomy in 28 (56%) respondents, cholecystectomy in 12 (24%) respondents, herniorrhaphy in 11 (22%) respondents, and trauma in four (8%) respondents. When asked about the current rate of C-sections observed in their communities, 44 (88%) described this procedure as either common or very common. Forty (80%) participants reported an overall preference among women in their community for delivery in a hospital setting, with only five (10%) reporting a community preference for home childbirth with the assistance of a midwife.

Though services through the public health system are subsidized by the Ministry of Public Health and Social Assistance (MSPAS), surgery at private hospitals was reported as “expensive” by all respondents, with estimated costs ranging from 3000-10000 Quetzals (US$384.60-US$1282.05). Access to surgery and anesthesia care was reported as inadequate by 46 (92%) of respondents. The greatest perceived barriers to care were costs in 35 (70%) respondents, travel/geographical distance in 22 (44%) respondents, and quality of care in 12 (24%) respondents.

## Discussion

In this study, we report the community perceptions of trends and barriers to surgical and anesthetic care in rural Southwest Guatemala. We identified significant utilization of anesthesia services among most surveyed households, and high demands for essential surgeries in participating communities. Yet, access was perceived to be inadequate by nearly all respondents. In this region, specialized surgical services are limited, and cost and geographic distance were reported as the primary barriers to essential surgical care [5]. 

There is growing demand for surgery worldwide, though the lowest surgical volumes have been reported in LMICs [10]. This results in significant unmet needs for essential surgeries, especially in rural areas with limited resources. In Guatemala, the population has increased by over 57% since the year 2000, and rural populations have increased to an estimated 8.5 million individuals in 2023 [11,12]. The public health sector provides coverage for the vast majority of the population. However, of the 83% of the population that receive subsidized or free care through the MSPAS program, a significant proportion have minimal access to these health services [13]. In Southwest Guatemala, communities have limited options for facilities and care within the public health system. Additionally, the unequal distribution of health worker density results in limited numbers of surgeons, obstetricians, and anesthesiologists in publicly funded hospitals in rural areas [6]. 

Common surgical conditions, such as appendicitis, cholecystitis, hernias, and trauma, were frequently reported among these rural communities. This trend has been observed in rural populations in other low- and middle-income countries [14]. Often referred to as the Bellwether procedures, the combination of cesarean delivery, open laparotomy, and treatment of open fractures has been recommended as a standard and a metric of local capabilities to provide essential surgery by the Lancet Global Commission [1,15]. Death from common surgical conditions, such as complications of pregnancy or abdominal emergencies, can be prevented by improved access to surgical care. For appendicitis alone, 38,000 deaths and 2 million disability adjusted life-years (DALYs) could be averted by essential surgery in low- and middle-income countries [16]. Of the 36 public hospitals that provide surgical care in Guatemala, nearly all have the ability to perform the three Bellwether procedures [6]. Although the closest regional hospital in the town of Coatepeque has the capabilities to perform these common surgeries, the capacity appears inadequate for the high demand among growing rural populations. While emergency surgery is often prioritized, elective surgery, such as herniorrhaphy, can be delayed for prolonged periods. This can result in significant health and economic consequences among the populations of agricultural workers in this Southwest region. Furthermore, for specialized surgical procedures, such as cardiac surgery, otolaryngology, neurosurgery, and transplant surgeries, referrals to the larger urban centers are required. Access to these specialized surgical centers may be complicated by travel times, prohibitive costs, and delays due to high demand.

In these communities of Southwest Guatemala, travel time to the nearest surgical center was a median of one hour, though a few respondents reported significantly longer times. One study of geospatial analysis determined that only 53.1% of individuals in Guatemala live within two hours of driving time to a surgical facility capable of performing essential surgeries, and that access varies greatly in large portions of the population [5]. While geographic analyses have been shown to underestimate real travel time, the reported distances among our cohort seem to be consistent with estimates [17]. The benchmark for timely access was defined by the Lancet Commission on Global Surgery (LCoGS) as a two-hour travel time to a center capable of emergency surgery [1]. However, the American College of Obstetricians and Gynecologists ACOG has proposed the “30-minute rule” for access to a facility capable of performing emergent C-sections, due to the higher risks associated with complicated deliveries [18]. The limitations of emergency medical services and public transportation from rural communities can compound challenges with accessing care within these recommended time intervals [19]. The geographic distance to obtain specialized surgery remains a significant barrier. Although Buda et al. report that the expansion of surgical services at existing hospitals in Guatemala would result in a minimal increase in overall geographic access, the authors ascertain that the expansion of surgical capabilities in Coatepeque in Southwest Guatemala would result in the highest increase in access nationwide and improve access for 123,868 people [5]. Moreover, the authors did not account for surgical specialties, which are severely limited and would benefit communities in this region. 

Cost was seen as the primary barrier, despite universal care through the public health system [6]. This may reflect strong preferences for care through the expensive private health system, indirect health costs, lodging and food, or transportation expenses. Although services and facilities through the public hospitals are typically free, the costs for medications and medical equipment are often passed on to patients, which can delay treatments if they are unable to be financed [20]. This finding is consistent with a prior survey of pediatric patients at a large urban hospital in Guatemala City who reported primary barriers related to financial costs and quality of care [21]. Likewise, financial restraints have commonly been cited as the primary barrier to care in other LMICs [14,22]. Multiple interview participants freely reported that they had incurred and would continue to opt for costly out-of-pocket expenditures to obtain services at private hospitals, due to a lack of confidence or delays in the public health system. Thus, efforts to strengthen the existing public health system and expand hospital capacity may improve perceived access to affordable care. 

Most surveyed participants reported having a direct family member who had undergone at least one Cesarean section. The perceived increasing rate of C-sections was a concern expressed by community members and is consistent with trends previously observed in this region and in other Latin American countries, where C-section rates are among the highest in the world [23-25]. Globally, C-sections comprise nearly one-third of all surgical volume in low health expenditure countries [10]. Optimizing the use of C-section through addressing unmet needs and reducing overuse is necessary to reduce the maternal and perinatal morbidity and mortality [26]. Rates of surgical deliveries above the WHO recommendations can lead to unnecessary risk and remain an active area of investigation and public health priority [27]. Furthermore, women in LMICs with a prior history of cesarean delivery are five times more likely to have a subsequent operative delivery [28]. This may partly explain the overwhelming preference among individuals surveyed for birth in hospital settings, as there is a significant portion of women with a history of prior operative deliveries in this region. Respondents cited safety concerns and risk of complications as common reasons for delivering in the hospital in this region. Due to limited care options, transportation issues, and inadequate emergency medical services, access to peripartum obstetric care, especially in emergency settings, is difficult for rural families and may contribute to the increased demand and utilization of inpatient obstetric and anesthesia services. 

Some initial strategies to improve access to care in this region could include improving quality and trust in the public health system, expanding the workforce and specialty capabilities of first referral hospitals, and minimizing indirect costs. Although the construction of new facilities would help reduce geographic distance to communities and expand surgical volume to meet the high demand, strengthening existing systems would result in a significant improvement in access in the Southwest region of Guatemala [5]. Alternative solutions that have been proposed include reducing cost and subsidizing care in private hospital facilities [1,5,29]. The expansion of emergency medical services in the area could also reduce preventable morbidity and mortality by expediting access to emergency care, though further evaluation of this strategy is warranted. Future efforts to reduce disparities and improve health system effectiveness and accessibility should integrate patient and community perspectives to ensure patient-centered approaches.

There are several limitations to this study. The small sample size constitutes a small proportion of the population in this region, which may not be representative of all neighboring communities and may limit the generalizability. Additionally, the sampling at a single health center may introduce selection bias, as patients who present for care at the TCHD may have different perspectives than those who do not seek professional health services. There is also an inherent risk of information bias with the cross-sectional study design, due to the potential for inaccurate reporting of community trends by participants. Furthermore, this study aims to examine the primary needs and barriers to accessing care, but does not explore other indicators, such as the decision to seek care or delays in receiving care, as outlined by the “three delays” framework [30]. Yet, the first-hand responses from individuals in this study provide valuable qualitative and descriptive data and offer important insight into the community experience and perceptions of needs and accessibility of perioperative services among these marginalized populations.

## Conclusions

Access to essential surgical services and anesthesia care is necessary to reduce global disparities in health. In rural Southwest Guatemala, there is significant utilization and demand for surgical and anesthetic care, particularly with the increasing rates of Cesarean sections. Community members report significant challenges to accessing care, such as cost, geographic distance, and quality of care. We discuss different strategies to promote increased access to care for the rural populations in this region. Further studies of community perceptions of local needs and barriers are needed to guide patient-centered interventions and policy for underrepresented rural populations.

## Appendices

Interview Guide

1.     Has anyone in your household undergone surgery and anesthesia?

¿Alguien en su hogar se ha sometido a cirugía y anestesia?

2.     If willing to share, what surgeries were performed?

Si desea compartir, ¿qué cirugías se realizaron?

3.     Where was the surgery performed?

¿Dónde se realizó la cirugía?

4.     For Cesarean sections, what was the method of anesthesia?

En el caso de las cesáreas, ¿cuál fue el método de anestesia?

5.     What is the most common surgery performed in your community?

¿Cuál es la cirugía más común que se realiza en su comunidad?

6.     What is the closest surgical hospital to your house?

¿Cuál es el hospital quirúrgico más cercano a su casa?

7.     How long would it take to get to the nearest surgical hospital?

¿Cuánto tiempo le tomaría llegar al hospital quirúrgico más cercano?

8.     Where would you go for emergency surgery?

¿A dónde iría para una cirugía de emergencia?

9.     Where would you go for specialized surgery?

¿A dónde iría para una cirugía especializada?

10.  Where do most women in your community deliver? Do most women prefer to deliver at home or in the hospital?

¿Dónde dan a luz la mayoría de las mujeres en su comunidad? ¿La mayoría de las mujeres prefieren dar a luz en casa o en el hospital?

11.  How common is Cesarean section among all deliveries?

¿Qué tan común es la cesárea entre todos los partos?

12.  Is there adequate access to anesthesia and surgery?

¿Hay acceso adecuado a la anestesia y la cirugía?

13.  What are the primary barriers to accessing care?

¿Cuáles son las principales barreras para acceder a la atención médica?

## References

[1] Meara JG, Leather AJ, Hagander L (2015). Global Surgery 2030: evidence and solutions for achieving health, welfare, and economic development. Lancet.

[2] Ologunde R, Maruthappu M, Shanmugarajah K, Shalhoub J (2014). Surgical care in low and middle-income countries: burden and barriers. Int J Surg.

[3] Kempthorne P, Morriss WW, Mellin-Olsen J, Gore-Booth J (2017). The WFSA Global Anesthesia Workforce Survey. Anesth Analg.

[4] Grimes CE, Bowman KG, Dodgion CM, Lavy CB (2011). Systematic review of barriers to surgical care in low-income and middle-income countries. World J Surg.

[5] Buda AM, Truche P, Izquierdo E (2022). Use of geospatial analysis for priority setting in surgical system investment in Guatemala. Lancet Reg Health Am.

[6] Zha Y, Truché P, Izquierdo E (2021). Assessment of anesthesia capacity in public surgical hospitals in Guatemala. Anesth Analg.

[7] von Elm E, Altman DG, Egger M, Pocock SJ, Gotzsche PC, Vandenbroucke JP (2007). The Strengthening the Reporting of Observational Studies in Epidemiology (STROBE) statement: guidelines for reporting observational studies. Lancet.

[8] Jimenez-Zambrano A, Avery M, Feller K (2024). Exploring the acceptability of a decision aid for rural women with a history of prior cesarean birth regarding subsequent mode of birth in Coatepeque, Guatemala. Front Glob Womens Health.

[9] Asturias EJ, Heinrichs G, Domek G (2016). The center for human development in Guatemala: an innovative model for global population health. Adv Pediatr.

[10] Weiser TG, Haynes AB, Molina G (2015). Estimate of the global volume of surgery in 2012: an assessment supporting improved health outcomes. Lancet.

[11] (2024). Rural population - Guatemala. https://data.worldbank.org/indicator/SP.RUR.TOTL?locations=GT.

[12] (2025). 2024 Health in the Americas: Guatemala - Country Profile. https://hia.paho.org/en/country-profiles/guatemala.

[13] Avila C, Bright R, Gutierrez J (2024). Guatemala Health System Assessment, August 2015. In Health Finance and Governance Project. https://www.slideshare.net/slideshow/guatemala-health-system-assessment-2015/58023480.

[14] Kalisya LM, Yap A, Mitume B, Salmon C, Karafuli K, Poenaru D, Onyango R (2023). Determinants of access to essential surgery in the Democratic Republic of Congo. J Surg Res.

[15] O'Neill KM, Greenberg SL, Cherian M (2016). Bellwether procedures for monitoring and planning essential surgical care in low- and middle-income countries: caesarean delivery, laparotomy, and treatment of open fractures. World J Surg.

[16] Mock CN, Donkor P, Gawande A, Jamison DT, Kruk ME, Debas HT (2015). Essential Surgery: Key Messages of This Volume.

[17] Rudolfson N, Gruendl M, Nkurunziza T (2020). Validating the global surgery geographical accessibility indicator: differences in modeled versus patient-reported travel times. World J Surg.

[18] Bank TC, Macones G, Sciscione A (2023). The "30-minute rule" for expedited delivery: fact or fiction?. Am J Obstet Gynecol.

[19] Juarez M, Austad K, Rohloff P (2021). Out-of-pocket costs for facility-based obstetrical care in rural Guatemala. Ann Glob Health.

[20] Bowser DM, Mahal A (2011). Guatemala: the economic burden of illness and health system implications. Health Policy.

[21] Nguyen K, Bhattacharya SD, Maloney MJ, Figueroa L, Taicher BM, Ross S, Rice HE (2013). Self-reported barriers to pediatric surgical care in Guatemala. Am Surg.

[22] Forrester JD, Forrester JA, Kamara TB (2016). Self-reported determinants of access to surgical care in 3 developing countries. JAMA Surg.

[23] Harrison MS, Scarbro S, Juarez-Colunga E (2019). Trends in the mode of delivery of pregnant women in rural Guatemala from a quality improvement database. Matern Child Health J.

[24] Betran AP, Ye J, Moller AB, Souza JP, Zhang J (2021). Trends and projections of caesarean section rates: global and regional estimates. BMJ Glob Health.

[25] Jimenez-Zambrano A, Feller K, Rivera C (2022). Perspectives of obstetricians and women with a history of prior cesarean birth regarding subsequent mode of birth in Trifinio and Coatepeque, Guatemala. Obstet Gynecol Res.

[26] Betrán AP, Temmerman M, Kingdon C (2018). Interventions to reduce unnecessary caesarean sections in healthy women and babies. Lancet.

[27] Betran AP, Torloni MR, Zhang JJ, Gülmezoglu AM (2016). WHO statement on caesarean section rates. BJOG.

[28] Figueroa L, Harrison M, Mazariegos M (2023). Maternal and perinatal outcomes of women with vaginal birth after cesarean section compared to repeat cesarean birth in select South Asian and Latin American settings of the global network for women's and children's health research. Matern Health Neonatol Perinatol.

[29] Jindal RM, Patel TG, Waller SG (2017). Public-private partnership model to provide humanitarian services in developing countries. J Am Coll Surg.

[30] Actis Danna V, Bedwell C, Wakasiaka S, Lavender T (2020). Utility of the three-delays model and its potential for supporting a solution-based approach to accessing intrapartum care in low- and middle-income countries. A qualitative evidence synthesis. Glob Health Action.

